# Shuidouchi (Fermented Soybean) Fermented in Different Vessels Attenuates HCl/Ethanol-Induced Gastric Mucosal Injury

**DOI:** 10.3390/molecules201119654

**Published:** 2015-10-30

**Authors:** Huayi Suo, Xia Feng, Kai Zhu, Cun Wang, Xin Zhao, Jianquan Kan

**Affiliations:** 1 College of Food Science, Southwest University, Chongqing 400715, China; birget@swu.edu.cn; 2 Chongqing Engineering Research Center of Regional Food, Chongqing 400715, China; 3 Department of Biological and Chemical Engineering, Chongqing University of Education, Chongqing 400067, China; fengxia@foods.ac.cn (X.F.); zhukai@foods.ac.cn (K.Z.); wangcun@foods.ac.cn (C.W.); 4 Chongqing Collaborative Innovation Center of Functional Food, Chongqing University of Education, Chongqing 400067, China

**Keywords:** vessel, Shuidouchi, gastric mucosal injury, antioxidant, mice

## Abstract

Shuidouchi (Natto) is a fermented soy product showing *in vivo* gastric injury preventive effects. The treatment effects of Shuidouchi fermented in different vessels on HCl/ethanol-induced gastric mucosal injury mice through their antioxidant effect was determined. Shuidouchi contained isoflavones (daidzein and genistein), and GVFS (glass vessel fermented Shuidouchi) had the highest isoflavone levels among Shuidouchi samples fermented in different vessels. After treatment with GVFS, the gastric mucosal injury was reduced as compared to the control mice. The gastric secretion volume (0.47 mL) and pH of gastric juice (3.1) of GVFS treated gastric mucosal injury mice were close to those of ranitidine-treated mice and normal mice. Shuidouchi could decrease serum motilin (MTL), gastrin (Gas) level and increase somatostatin (SS), vasoactive intestinal peptide (VIP) level, and GVFS showed the strongest effects. GVFS showed lower IL-6, IL-12, TNF-α and IFN-γ cytokine levels than other vessel fermented Shuidouchi samples, and these levels were higher than those of ranitidine-treated mice and normal mice. GVFS also had higher superoxide dismutase (SOD), nitric oxide (NO) and malonaldehyde (MDA) contents in gastric tissues than other Shuidouchi samples. Shuidouchi could raise IκB-α, EGF, EGFR, nNOS, eNOS, Mn-SOD, Gu/Zn-SOD, CAT mRNA expressions and reduce NF-κB, COX-2, iNOS expressions as compared to the control mice. GVFS showed the best treatment effects for gastric mucosal injuries, suggesting that glass vessels could be used for Shuidouchi fermentation in functional food manufacturing.

## 1. Introduction

Soybeans fermented in water for a short time (Shuidouchi) is a traditional Chinese fermented soybean product, whose process is similar to Chungjukjang from South Korea and Natto from Japan [1]. It rich in nutrients, including proteins, vitamins and minerals, and its content of vitamin E is especially noteworthy [2]. Fermented soybeans not only have high nutritive value, but are also used as a drug in Traditional Chinese Medicine [3]. According to Traditional Chinese Medicine, fermented soybeans can clear heat and detoxify, which can treat headaches due to pathogenic wind-heat, chest distress and vomiting. Numerous soy oligosaccharides in fermented soybeans can improve the body’s immunity and reduce the intestinal levels of toxic substances, which can prevent intestinal tumors [4]. Scholars in Japan and South Korea found that Chungjukjang and Natto have many health benefits, including anti-oxidation, anti-inflammatory and anti-cancer activities. As a soy product, the most active ingredient of soybeans fermented in water is soy isoflavones. Isoflavones in fermented soybean products are more active than that in raw soybeans, which have very strong anti-tumor and anti-aging effects and prevent embrittlement of blood capillaries [5].

Shuidouchi is a soy product fermented for a short time [6]. In addition to factories, it can also be homemade in many areas. Similarly, in Japan and South Korea, they often make Natto and Chungkukjang at home. Different kinds of containers are often used in fermentation of Natto and Chungkukjang both in the factories and at home. Japan has even developed an automatic Natto fermentation machine with a metal tank. South Korean scholars have studied the sensory, physical and chemical properties as well as antioxidant effects of Chungkukjang fermentation with jars and glass, they found that jars were much better for the production of Chungkukjang [7]. In China, metal, glass, plastic and ceramic containers are often used in Shuidouchi production. This research aims to study the anti-gastric mucosal injury effects of Shuidouchi fermented in different containers and explain the mechanism of the anti-oxidation effects of Shuidouchi.

The stomach is in a protected anatomical position in abdominal cavity and can move within some limits, so it is not easy to injure it with outside violence [8]. Gastric mucosal damage is very common and caused by many factors, including chemical factors such as smoking, drinking strong tea, coffee, and drugs stimulating the gastric mucosa such as aspirin and indomethacin, physical factors such as too much cold or heat, eating rough food, bacteria or their toxins [9]. The alcohol in wine can greatly stimulate the gastric mucosa, and taking in too much alcohol can lead to gastric mucosa damage and congestion in the stomach [10]. Most bean products can protect the stomach, as they take advantage of their alkaline characteristics to neutralize the amount of hydrochloric acid due to gastric damage and alleviate stomach injuries. In addition to their alkalinity, soy isoflavones in soybean products may play a key role in alleviating stomach damage [2]. Soybean foods contain many isoflavones, such as daidzein, genistein, glycitein, *etc.*, but these isolflavones cannot be immediate absorbed in the human body, and they must be hydrolysed to absorbable aglycones by β-glucosidase from the intestinal microbiota. Shuidouchi is produced by microorganisms and these microorganism could make these isoflavones change into functional compounds which could be readily absorbed by humans.

A mice model of gastric mucosal injury induced by hydrochloric acid and alcohol can determine the health effects of functional food components. Alcohol is the main factor causing gastric mucosal injury, and a certain concentration of hydrochloric acid can promote and increase gastric mucosa lesions caused by alcohol. Based on this animal model, mice are gavaged with soybean isoflavones extracted from fermented soybeans to test biochemical indicators of serum levels (MTL, Gas, SS, VIP, IL-6, IL-12, TNF-α and IFN-γ), tissue levels (SOD, NO and MDA) and mRNA expression (NF-κB, IκB-α, EGF, EGFR, nNOS, eNOS, iNOS, COX-2, Mn-SOD, Gu/Zn-SOD and CAT) in gastric tissues. The experimental results prove the gastric mucosa damage prevention effects of isoflavones in fermented soybeans and help elucidate its possible mechanism. This study also aimed to know the physicochemical properties of Shuidouchi produced by fermentation in different vessels, and the relationship between the physicochemical properties discrepancies (isoflavones content, moisture content, fermentation temperature, acidity and total bacterial count) and anti-gastric mucosa damage effects.

## 2. Results and Discussion

### 2.1. Isoflavone Contents of Shuidouchi

In this study, the isoflavone contents of Shuidouchi was determined by a spectrophotometric method, whereby isoflavone standard solutions (daidzein and genistein) were measured, and the regression equation of contents of daidzein and genistein were made, Y_daidzein_ (daidzein regression equation) = 148338 + 1.70 × 10^8^ × (*r* = 0.999), Y_genistein_ (genistein regression equation) = −316706 + 4.20 × 10^8^ × (*r* = 0.995).

Compared with these regression equations, the results showed that GVFS has the highest contents of daidzein and genistein (Table 1), and MVFS had more daidzein and genistein content than PVFS and CVFS (*p* < 0.05). Soybean isoflavones can prevent and cure many diseases [11]. Isoflavones are limited in Nature and soybean is the only nutritionally-meaningful food source of isoflavones [12]. Many studies had shown that soybean isoflavones have strong antioxidant effects, especially the soybean isoflavones in the human body, which have strong antioxidant and anti-inflammatory effects [13,14,15]. Recent research showed that the content of soybean isoflavones is higher in Shuidouchi, and the content was much higher in fermented soybeans because of the fermentation effect [2]. 

During the fermentation process, many factors could affect the contents of isoflavones in Shuidouchi, including the fermentation container type. Temperature and moisture are important factors in fermentations. By temperature control, the inside and outside of the container could remain unobstructed, which was advantageous for natural fermentation, while adequate moisture was also helpful for fermentation [5]. Diathermancy and moisture retention of glass containers were better than the other vessels, which were more advantageous to Shuidouchi fermentation [16]. They could produce more soybean isoflavones, which might inhibit gastric lesions.

**Table 1:** Contents of soybean isoflavones in different vessels fermented Shuidouchi.

Group	Daidzein (mg/g)	Genistein (mg/g)
CVFS	0.45 ± 0.03 ^c^	0.72 ± 0.04 ^c^
PVFS	0.48 ± 0.04 ^c^	0.73 ± 0.03 ^c^
MVFS	0.67 ± 0.02 ^b^	0.96 ± 0.04 ^b^
GVFS	0.84 ± 0.03 ^a^	1.22 ± 0.05 ^a^

^a–c^ Mean values with different letters in the same column are significantly different (*p* < 0.05) according to Duncan’s multiple-range test. CVFS, ceramic vessel fermented Shuidouchi; PVFS, plastic vessel fermented Shuidouchi; MVFS, metal vessel fermented Shuidouchi; GVFS, glass vessel fermented Shuidouchi.

### 2.2. Physicochemical Properties of Shuidouchi

After 72 h fermentation, the moisture content, temperature, acidity and total bacterial counts of Shuidouchi samples fermented in different vessels were determined. GVFS had the highest moisture content and total bacterial counts, but it had a lower temperature than CVFS, PVFS, MVFS and had lower acidity than CVFS, PVFS (Table 2).

**Table 2:** Physicochemical properties of Shuidouchi fermented in different vessels.

Group	Moisture Content (%)	Temperature (°C)	Acidity (%)	Total Bacterial Counts (×10^9^ CFU/g)
CVFS	53.82 ± 0.03 ^d^	38.84 ± 0.08 ^b^	0.92 ± 0.07 ^a^	1.03 ± 0.07 ^d^
PVFS	54.10 ± 0.02 ^c^	39.72 ± 0.21 ^a^	0.89 ± 0.06 ^a^	1.24 ± 0.06 ^c^
MVFS	56.39 ± 0.04 ^b^	37.36 ± 0.12 ^c^	0.81 ± 0.09 ^ab^	1.56 ± 0.06 ^b^
GVFS	57.37 ± 0.01 ^a^	37.42 ± 0.13 ^c^	0.72 ± 0.11 ^b^	1.78 ± 0.12 ^a^

^a–d^ Mean values with different letters in the same column are significantly different (*p* < 0.05) according to Duncan’s multiple-range test. CVFS, ceramic vessel fermented Shuidouchi; PVFS, plastic vessel fermented Shuidouchi; MVFS, metal vessel fermented Shuidouchi; GVFS, glass vessel fermented Shuidouchi.

Moisture is a essential factor for bacterial growth, as the richness of moisture favors the proliferation of bacteria, so rich moisture could help Shuidouchi to ferment [17]. Glass vessels could retain the moisture during the fermentation of Shuidouchi, which might promote this fermentation and make GVFS a high quality soybean fermented food. Shuidouchi was fermented at 36 °C in this study, and the temperatures of Shuidouchi changed in the different vessels. Under the same temperature conditions, these changes result from the heat conductivities of different vessels. The heat conductivities of glass and metal are better than those of ceramic and plastic. Thirty six (36) °C is a suitable temperature for bacterial growth [18], the fermentation temperature of glass and metal vessels fermented Shuidouchi were close to 36 °C, and glass and metal vessels could maintain a suitable temperature for Shuidouchi fermentation. The high acidity could inhibit the bacteria growth, and maintaining a convenient acidity would help bacterial growth [19]. Glass vessels can keep a convenient acidity for fermentation of Shuidouchi. From the results, glass vessels provide a better fermentation environment for the fermentation of Shuidouchi than other vessels, as glass vessels could help the Shuidouchi have more moisture content, total bacterial counts and lower temperature, acidity, and these conditions could make GVFS produce more daidzein and genistein than Shuidouchi fermented in other vessels.

### 2.3. Stomach Appearances of Mice 

**Figure 1:**
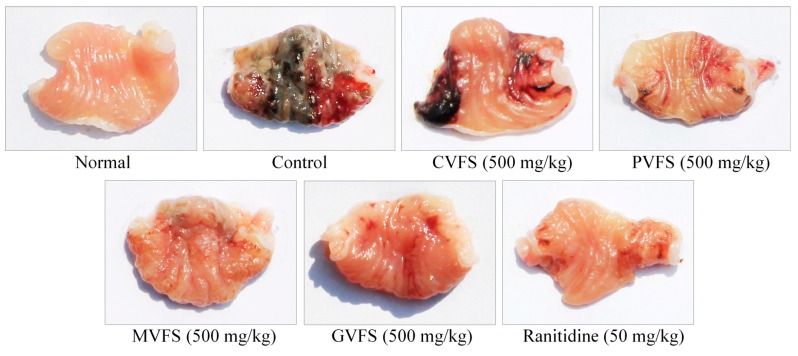
Stomachs with HCl/ethanol-induced gastric mucosal injury in mice treated with Shuidouchi fermented in different vessels. CVFS—ceramic vessel fermented Shuidouchi; PVFS—plastic vessel fermented Shuidouchi; MVFS—metal vessel fermented Shuidouchi; GVFS—glass vessel fermented Shuidouchi.

After Shuidouchi treatment, the gastric mucosal injury areas were reduced as compared to the control mice (Table 3, Figure 1), and the gastric mucosal injury area of GVFS-treated mice was significantly (*p* < 0.05) lower from other vessel fermented Shuidouchi-treated mice. The area was close to that of the ranitidine-treated mice. The inhibitory rate of GVFS-treated mice was also higher than that of other Shuidouchi-treated mice.

**Table 3:** Stomach appearance of HCl/ethanol induce gastric mucosal injury mice treated with Shuidouchi fermented in different vessels.

Group	Gastric Mucosal Injury	
	Gastric Mucosal Injury Area (mm^2^)	Inhibitory Rate (%)
Normal	0.0 ± 0.0 ^f^	100 ± 0.0 ^a^
Control	7.21 ± 0.62 ^a^	0.0 ± 0.0 ^d^
CVFS (500 mg/kg)	2.88 ± 0.36 ^b^	60.1 ± 4.6 ^e^
PVFS (500 mg/kg)	2.75 ± 0.31 ^b^	61.9 ± 4.1 ^b^
MVFS (500 mg/kg)	2.15 ± 0.18 ^c^	70.2 ± 2.5 ^d^
GVFS (500 mg/kg)	1.84 ± 0.21 ^d^	74.5 ± 2.7 ^c^
Ranitidine (50 mg/kg)	1.05 ± 0.17 ^e^	85.4 ± 2.4 ^b^

^a–f^ Mean values with different letters in the same column are significantly different (*p* < 0.05) according to Duncan’s multiple-range test. CVFS, ceramic vessel fermented Shuidouchi; PVFS, plastic vessel fermented Shuidouchi; MVFS, metal vessel fermented Shuidouchi; GVFS, glass vessel fermented Shuidouchi.

After administering HCl/ethanol, the gastric mucosal injury area, an important index for gastric mucosal injury determination, could be increased, [20]. From this study, GVFS could decrease the HCl/ethanol induced gastric mucosal injury, and its effects seem stronger than those of Shuidouchi fermented in other vessels.

### 2.4. Gastric Secretion Volume and pH of Gastric Juice of Mice 

GVFS-treated mice had the lower gastric secretion volume but higher than that of normal and drug (ranitidine)-treated mice (Table 4). GVFS-treated mice also had higher gastric juice pH than mice treated with Shuidouchi fermented in other vessels, close to the normal and ranitidine-treated mice.

**Table 4:** Gastric secretion volume and pH of gastric juice of HCl/ethanol induced gastric mucosal injury mice treated with Shuidouchi fermented in different vessels.

Group	Gastric Secretion Volume (mL)	pH of the Gastric Juice
Normal	0.28 ± 0.03 ^f^	3.6 ± 0.1 ^a^
Control	1.12 ± 0.17 ^a^	1.0 ± 0.2 ^f^
CVFS (500 mg/kg)	0.71 ± 0.11 ^b^	2.4 ± 0.4 ^e^
PVFS (500 mg/kg)	0.68 ± 0.12 ^b^	2.5 ± 0.3 ^e^
MVFS (500 mg/kg)	0.57 ± 0.05 ^c^	2.8 ± 0.2 ^d^
GVFS (500 mg/kg)	0.47 ± 0.04 ^d^	3.1 ± 0.1 ^c^
Ranitidine (50 mg/kg)	0.38 ± 0.04 ^e^	3.3 ± 0.2 ^b^

^a–f^ Mean values with different letters in the same column are significantly different (*p* < 0.05) according to Duncan’s multiple-range test. CVFS, ceramic vessel fermented Shuidouchi; PVFS, plastic vessel fermented Shuidouchi; MVFS, metal vessel fermented Shuidouchi; GVFS, glass vessel fermented Shuidouchi.

After determination of gastric secretion volume and pH of gastric juice, the gastric mucosal injuries could be checked out. High gastric secretion volume and low pH of the gastric juice cause severe gastric mucosal injury [21]. Shuidouchi could reduce the gastric secretion volume and raise the pH of the gastric juice, and the GVFS had the most obvious effects. 

### 2.5. Serum Motilin (MTL), Gastrin (Gas), Somatostatin (SS) and Vasoactive Intestinal Peptide (VIP) Levels in Mice 

GVFS could significantly (*p* < 0.05) increase the serum SS, VIP levels and decrease MTL, Gas levels compared to the control mice (Table 5). The serum MTL, Gas levels of GVFS treated mice were lower than with Shuidouchi fermented in other vessels, and serum SS, VIP levels of GVFS-treated mice were higher than those of animals treated with Shuidouchi fermented in other vessels. These levels were close to those of the ranitidine-treated mice and normal mice.

MTL is the gastrointestinal hormone of excitability. After stimulation, its content increases and this case abundant secretion of hydrochloric acid, which makes the stomach acidic and worsens the degree of gastric ulceration [22]. Stimulated by certain substances, Gas would be released into the blood and stimulate the parietal cells to secrete hydrochloric acid. The abnormal secretion of gastric acid worsens gastric mucosal injuries [23]. SS was not only a kind of neural hormone, but also a kind of neuromodulator, which could inhibit the secretion of many gastrointestinal hormones, reduce gastrointestinal peristalsis, blood flow in the viscera and portal veins, as well as release of inflammatory mediators, thus inhibiting gastric mucosal injuries [24]. VIP is a gastrointestinal inhibitory hormone, which could inhibit the secretion of stomach acid. Meanwhile, VIP could activate the D cells on the stomach wall. D cells release somatostatin, which could inhibit the secretion of gastrin by G cells on the stomach wall and play a key role of alleviating gastric mucosa injuries [25].

**Table 5:** Serum MTL, Gas, SS and VIP levels of HCl/ethanol induce gastric mucosal injury mice treated with Shuidouchi fermented in different vessels. Define all in table caption.

Group	MTL (μg/L)	Gas (μg/L)	SS (μg/L)	VIP (μg/L)
Normal	42.0 ± 2.3 ^f^	67.6 ± 5.3 ^f^	128.0 ± 5.3 ^a^	99.8 ± 4.3 ^a^
Control	108.3 ± 6.9 ^a^	137.6 ± 7.9 ^a^	74.1 ± 3.3 ^f^	51.2 ± 2.9 ^f^
CVFS (500 mg/kg)	73.2 ± 5.2 ^b^	95.4 ± 4.8 ^b^	95.4 ± 7.8 ^e^	70.3 ± 3.6 ^e^
PVFS (500 mg/kg)	70.6 ± 4.7 ^b^	90.6 ± 6.1 ^b^	97.6 ± 4.6 ^e^	74.1 ± 3.3 ^e^
MVFS (500 mg/kg)	59.7 ± 4.5 ^c^	85.1 ± 3.6 ^c^	105.3 ± 3.0 ^d^	80.1 ± 2.5 ^d^
GVFS (500 mg/kg)	52.6 ± 3.1 ^d^	77.3 ± 3.2 ^d^	114.1 ± 3.8 ^c^	86.7 ± 2.8 ^c^
Ranitidine (50 mg/kg)	47.6 ± 2.0 ^e^	72.5 ± 2.2 ^e^	120.6 ± 4.8 ^b^	92.5 ± 3.1 ^b^

^a–f^ Mean values with different letters in the same column are significantly different (*p* < 0.05) according to Duncan’s multiple-range test. CVFS, ceramic vessel fermented Shuidouchi; PVFS, plastic vessel fermented Shuidouchi; MVFS, metal vessel fermented Shuidouchi; GVFS, glass vessel fermented Shuidouchi.

### 2.6. Cytokine IL-6, IL-12, TNF-α and IFN-γ Levels in Mice 

Cytokine IL-6, IL-12, TNF-α and IFN-γ levels in normal mice were the lowest, and these levels in control mice were the highest (Table 6). GVFS-treated mice showed a lower level than control mice and mice treated with Shuidouchi fermented in other vessels, and only higher than ranitidine-treated mice and normal mice.

**Table 6:** Cytokine IL-6, IL-12, TNF-α and IFN-γ levels of HCl/ethanol induce gastric mucosal injury mice treated with Shuidouchi fermented in different vessels.

Group	IL-6 (pg/mL)	IL-12 (pg/mL)	TNF-α (pg/mL)	IFN-γ (pg/mL)
Normal	41.2 ± 2.6 ^g^	278.6 ± 33.5 ^g^	43.5 ± 1.7 ^f^	44.2 ± 1.5 ^f^
Control	119.3 ± 7.3 ^a^	942.1 ± 42.1 ^a^	125.6 ± 8.3 ^a^	99.2 ± 5.1 ^a^
CVFS (500 mg/kg)	71.6 ± 4.8 ^b^	651.2 ± 32.5 ^b^	75.1 ± 6.2 ^b^	68.7 ± 3.3 ^b^
PVFS (500 mg/kg)	65.1 ± 2.5 ^c^	608.6 ± 28.7 ^c^	73.5 ± 6.8 ^b^	65.3 ± 4.1 ^b^
MVFS (500 mg/kg)	57.5 ± 3.3 ^d^	506.7 ± 35.7 ^d^	60.6 ± 4.9 ^c^	57.1 ± 2.6 ^c^
GVFS (500 mg/kg)	50.3 ± 2.3 ^e^	425.6 ± 40.4 ^e^	55.6 ± 3.8 ^d^	51.2 ± 1.9 ^d^
Ranitidine (50 mg/kg)	45.3 ± 1.9 ^f^	361.5 ± 31.8 ^f^	49.2 ± 2.8 ^e^	47.6 ± 1.6 ^e^

^a–g^ Mean values with different letters in the same column are significantly different (*p* < 0.05) according to Duncan’s multiple-range test. CVFS, ceramic vessel fermented Shuidouchi; PVFS, plastic vessel fermented Shuidouchi; MVFS, metal vessel fermented Shuidouchi; GVFS, glass vessel fermented Shuidouchi.

IL-6 is a cytokine secreted by T cells, B cells and mononuclear macrophages, which becomes abnormal in many autoimmune diseases and is related to the pathological process and severity of these diseases [26]. A variety of abnormal antibodies and immune complexes in patients with gastric injuries could stimulate the monocyte-macrophages to produce and release TNF-α into blood circulation through different ways, causing increases of TNF-α levels in blood. The interaction between increased TNF-α and inflammatory cells worsens the inflammation and promotes gastric mucosa damage. IL-12 and IFN-γ are pro-inflammatory cytokines [27].

IL-12 could promote the growth and proliferation of T cells and NK cells and stimulate these cells to produce IFN-γ, which could increase the secretion of IL-12 [28]. Research had shown that IFN-γ and IL-12 could take part in gastric mucosa injuries and treatment through different interactions [29]. Under laboratory conditions, reducing the level of IL-6, IL-12, TNF-α and IFN-γ could reduce the degree of gastric injury in mice. Soy isoflavones in fermented soybeans water alleviated the effect of gastric mucosa injury by lowering the level of IL-6, IL-12, TNF-α and IFN-γ in serum [25]. 

### 2.7. Gastric Tissue SOD, NO and MDA Activities of Mice 

After the gastric tissues determination, GVFS-treated mice showed higher SOD, NO contents than PVFS-, MVFS-, and CVFS-treated mice and control mice (Table 7). Control mice had the highest MDA content in gastric tissue, Shuidouchi could reduce the MDA content in gastric tissue, and GVFS decreased the MDA content the most as compared to Shuidouchi fermented in other vessels.

**Table 7:** Gastric tissues SOD, NO and MDA activities of HCl/ethanol induce gastric mucosal injury mice treated with Shuidouchi fermented in different vessels.

Group	SOD (kU/L)	NO (μmol/L)	MDA (μmol/L)
Normal	379.4 ± 41.2 ^a^	14.5 ± 0.3 ^a^	20.5 ± 1.7 ^f^
Control	107.9 ± 23.5 ^g^	3.1 ± 0.2 ^f^	84.1 ± 4.3 ^a^
CVFS (500 mg/kg)	210.6 ± 34.3 ^f^	7.5 ± 0.4 ^e^	48.7 ± 4.6 ^b^
PVFS (500 mg/kg)	237.6 ± 33.1 ^e^	7.9 ± 0.5 ^e^	47.9 ± 4.4 ^b^
MVFS (500 mg/kg)	271.3 ± 31.0 ^d^	9.3 ± 0.4 ^d^	35.1 ± 2.9 ^c^
GVFS (500 mg/kg)	303.5 ± 27.6 ^c^	11.2 ± 0.5 ^c^	30.1 ± 2.5 ^d^
Ranitidine (50 mg/kg)	341.2 ± 25.6 ^b^	12.7 ± 0.3 ^b^	26.4 ± 2.3 ^e^

^a–g^ Mean values with different letters in the same column are significantly different (*p* < 0.05) according to Duncan’s multiple-range test. CVFS, ceramic vessel fermented Shuidouchi; PVFS, plastic vessel fermented Shuidouchi; MVFS, metal vessel fermented Shuidouchi; GVFS, glass vessel fermented Shuidouchi.

NO in the body is generated by NOS catalysis, which has cytotoxic effects and is involved in mediating immune reactions [30]. In recent years, studies have shown that many pathological stomach disease processes are associated with abnormal changes of NO levels [30,31]. As the only synthetase of NO, the distribution and function of NOS in the stomach is a hotspot of current research. NO could protect the gastric mucosa, which is one of its main functions in the stomach. It was widely accepted that NO mediates gastric mucosa to produce prostaglandins and increase the blood flow of gastric mucosa to protect it [32]. It was generally agreed that the effect of NO on gastric motility is an inhibitory process, and NO has a diastolic function and could delay gastric emptying ability. It has been found that a large number of NOS exist in gastric smooth cells and between the gastric muscle nerve plexus, which shows that NO plays an important role in regulating gastric motility [33]. When gastritis happens, the content of NO in stomach tissues would drop significantly, while this study also showed the same result [34].

Oxygen free radicals are a kind of oxygenic gene with high chemical activity and produced by oxygen metabolism. As inflammatory mediators, free radicals were closely related with gastritis and also a very important initiation factor and independent pathogenic factor of gastric mucosal injury [35]. Under normal circumstances, SOD is a protection factor of gastric mucosal cells, which could remove oxygen free radicals and resist lipid peroxidation of the gastric mucosa epithelium in order to keep oxygen free radicals at a low level and avoid gastric mucosal epithelial cell damage. However, under pathological conditions, the body produces a large number of oxygen free radicals by enzyme and/or non-enzyme systems [36]. These free radicals could attack polyunsaturated fatty acids in the phospholipids of biological membranes and can cause lipid peroxidation, which might lead to biological membrane damage, protein denaturation, DNA damage, gene aberration and cell necrosis [37]. MDA is the final product of lipid peroxides, so the level of MDA can reflect the level of oxygen free radicals. Under normal circumstances, the free radical removing system in the human body could effectively decompose the free radicals to avoid too much MDA and cause no harm to the body, but if the stomach is injured, there are too many free radicals, or the defense system for removing free radicals fails, this could cause free radical damage to the body and too much MDA [38].

### 2.8. mRNA Expression of NF-κB, IκB-α, EGF and EGFR in Gastric Tissues of Mice 

**Figure 2:**
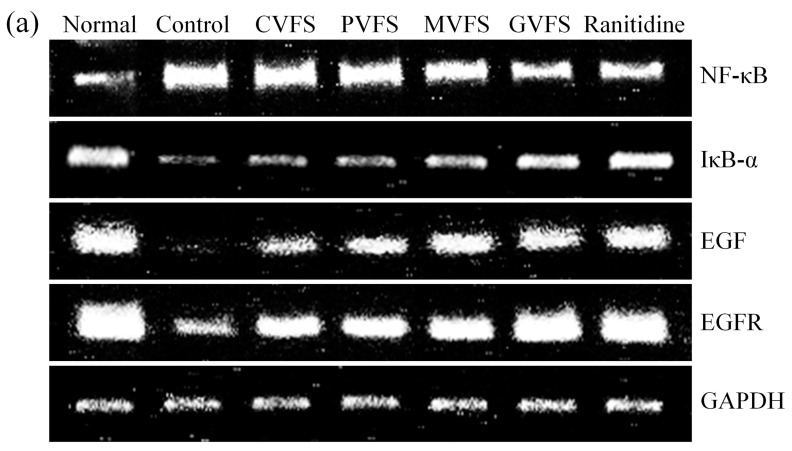

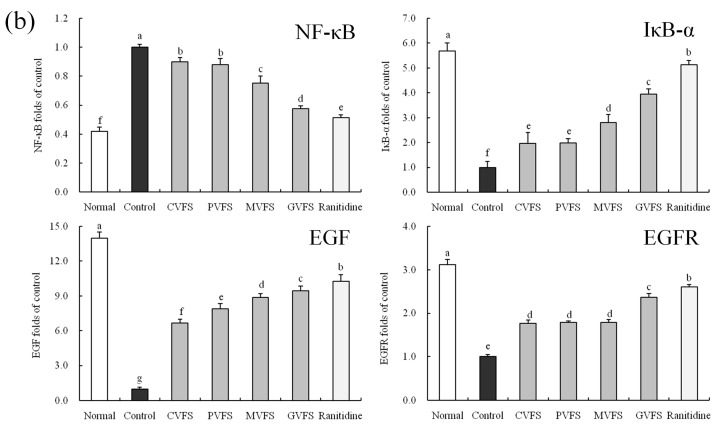
Effect of Shuidouchi fermented in different vessels on the NF-κB, IκB-α, EGF and EGFR mRNA expression in HCl/ethanol induced gastric mucosal injury mice. (**a**) bands of mRNA gene expression; (**b**) quantitative and ratio analysis. Fold-ratio: gene expression/GAPDH × control numerical value (control fold ratio: 1). ^a–g^ Mean values with different letters over the bars are significantly different (*p* < 0.05) according to Duncan’s multiple range test. CVFS, 500 mg/kg ceramic vessel fermented Shuidouchi; PVFS, 500 mg/kg plastic vessel fermented Shuidouchi; MVFS, 500 mg/kg metal vessel fermented Shuidouchi; GVFS, 500 mg/kg glass vessel fermented Shuidouchi; Ranitidine, 50 mg/kg ranitidine.

By the RT-PCR assay, the results showed that Shuidouchi could decrease the NF-κB mRNA expressions while increase the IκB-α and EGF. EGFR expressions of gastric tissues as compared to the control mice (Figure 2). In the gastric tissues of GVFS-treated mice, the NF-κB, IκB-α, EGF and EGFR were at 0.57, 3.95, 9.46 and 2.37 fold the levels of control mice, close to those of drug (ranitidine)-treated mice and normal mice. 

EGFR is proliferation and signal transduction receptor of EGF cells [39]. Studies have proven that many solid tumors show high or abnormal expression of EGFR, while appropriate EGFR and EGF is helpful to alleviate stomach injury [40,41]. Besides, EGF could inhibit gastric acid secretion, increase the secretion of gastric juice, increase mucous membrane blood flow, and protect the mucous membrane. All these functions are mainly mediated by EGFR. The regulation mechanism of EGF synthesis and release is very complicated [42]. It was reported that androgen can increase EGF synthesis in mice submandibular glands, while VIP could promote the salivary glands to secret EGF. As a receptor, the regulation of EGFR was more complex, but the expression of EGF and EGFR are positively correlated when the stomach is injured [43].

### 2.9. mRNA Expression of nNOS, eNOS, iNOS and COX-2 in Gastric Tissues of Mice 

**Figure 3:**
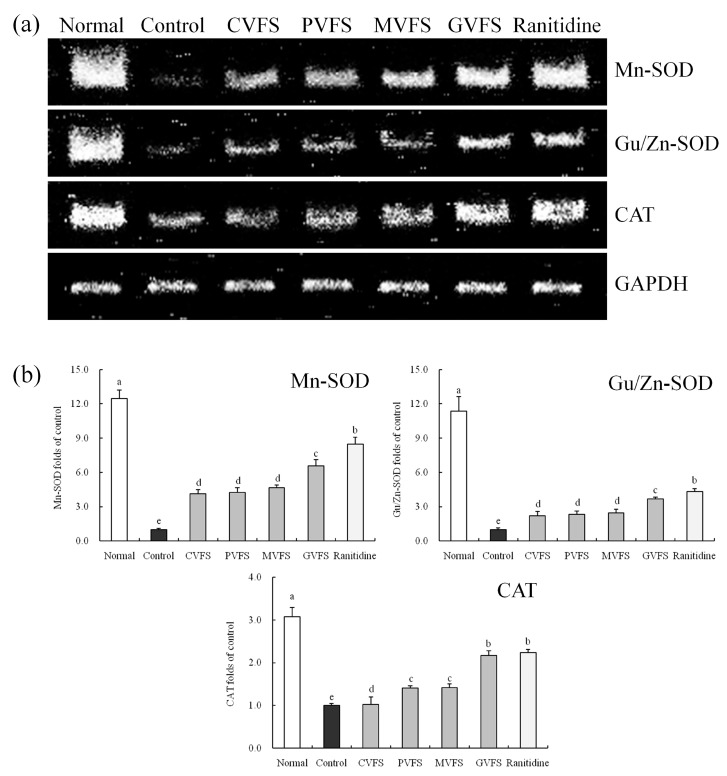
Effect of Shuidouchi fermented in different vessels on the nNOS, eNOS, iNOS and COX-2 mRNA expression in HCl/ethanol induced gastric mucosal injury mice. (**a**) bands of mRNA gene expression; (**b**) quantitative and ratio analysis. Fold-ratio: gene expression/GAPDH × control numerical value (control fold ratio: 1). ^a–e^ Mean values with different letters over the bars are significantly different (*p* < 0.05) according to Duncan’s multiple range test. CVFS, 500 mg/kg ceramic vessel fermented Shuidouchi; PVFS, 500 mg/kg plastic vessel fermented Shuidouchi; MVFS, 500 mg/kg metal vessel fermented Shuidouchi; GVFS, 500 mg/kg glass vessel fermented Shuidouchi; Ranitidine, 50 mg/kg ranitidine.

GVFS had higher nNOS (2.36-fold the control group), eNOS (2.05-fold the control group) mRNA expressions and lower iNOS (0.24-fold the control group), COX-2 (0.16-fold the control group) expressions than Shuidouchi fermented in other vessels in gastric tissues of mice (Figure 3). The nNOS, eNOS expressions of GVFS-treated mice were also significantly (*p* < 0.05) higher than control mice, while the iNOS, COX-2 expressions were lower than in control mice.

Normal endothelial cells have anticoagulant, anti-inflammatory, and angiogenesis inhibiting functions and also promote vasodilatation by generating NO, prostacyclin and other vasodilator substances [44]. Endothelial dysfunction could cause stomach inflammation, and lower bioavailability of NO is an important factor of endothelial dysfunction. NO is synthesized by NOS catalysis. NOS has three different subtypes, including NOS1 (nNOS), NOS2 (iNOS) and NOS3 (eNOS).

Under normal physiological conditions, NO in vascular endothelial cells mainly came from eNOS to regulate normal physiological functions [45], but under some pathological conditions, eNOS shows dysfunction, generating O_2_^−^ instead of NO, which decreases the bioavailability of NO and increases the oxidative stress, causing or aggravating endothelial dysfunction [46]. In a rest state, iNOS doesn’t express, but under pathological conditions, a large amount of iNOS and NO are produced. NO plays a dual role in inflammation. After activation for 4 to 6 h, iNOS could produce a lot of NO. In this study, the content of NO fell in the early stages of stomach injury and might increase continuously. In the early stages of inflammation, iNOS increases sharply and turns into NO after 4 to 6 h [47]. Excessive NO could intensify any gastric mucosa damage and activate COX-2 by combining with COX-2 at its active site, resulting in aggravated inflammation [48]. eNOS and nNOS are both Ca^2+^-dependent NOS, whose expression always shows a positive correlation in stomach tissues. When the gastric mucosa is damaged, the expression of NOS decreases, so does the expression of nNOS [49]. iNOS and COX-2 which are also important expressors of inflammation. Inflammation caused by stomach mucosa damage increases the expression of iNOS and COX-2, which is observed in both human clinical experiments and animal experiments. Stomach tissues of control group mice with gastric damage also showed the same expression [50]. Soy isoflavones from soybeans fermented in glass vessels significantly alleviate the phenomenon, and with the increase of its concentration, the alleviating effect was enhanced.

### 2.10. mRNA Expression of Mn-SOD, Gu/Zn-SOD and CAT in Gastric Tissues of Mice 

**Figure 4:**
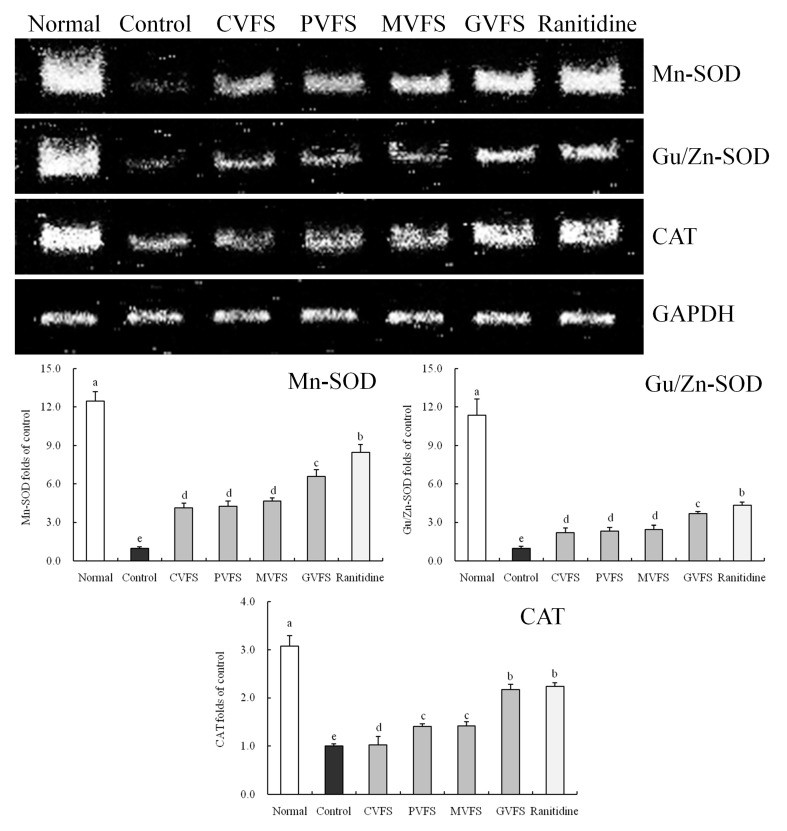
Effect of Shuidouchi fermented in different vessels on the Mn-SOD, Gu/Zn-SOD and CAT mRNA expression in HCl/ethanol induced gastric mucosal injury mice. (**a**) bands of mRNA gene expression; (**b**) quantitative and ratio analysis. Fold-ratio: gene expression/GAPDH × control numerical value (control fold ratio: 1). ^a–e^ Mean values with different letters over the bars are significantly different (*p* < 0.05) according to Duncan’s multiple range test. CVFS, 500 mg/kg ceramic vessel fermented Shuidouchi; VFS, 500 mg/kg plastic vessel fermented Shuidouchi; MVFS, 500 mg/kg metal vessel fermented Shuidouchi; GVFS, 500 mg/kg glass vessel fermented Shuidouchi; Ranitidine, 50 mg/kg ranitidine. Before sending to publish, try your best to move the caption in one page.

After inducing the gastric mucosal injury (control group), the mRNA expressions of Mn-SOD, Gu/Zn-SOD and CAT were significantly (*p* < 0.05) reduced as compared to normal mice (Figure 4). Shuidouchi could retard these reductions, and GVFS had the best effects. The Mn-SOD (6.60-fold of control group), Gu/Zn-SOD (3.68-fold of control group) and CAT (2.17-fold of control group) expressions of gastric tissues in GVFS-treated mice were the highest among the Shuidouchi-treated mice, and these expressions were close to those of ranitidine-treated mice and normal mice.

SOD has three kinds of isomers in animals, including Cu/Zn-SOD, Mn-SOD and EC-SOD (SOD3), while Cu/Zn-SOD and Mn-SOD are the two main types of SODs, which are antioxidant enzymes in the body [51]. Extreme decreases of Cu/Zn-SOD and Mn-SOD in the body imply the production of large numbers of free radicals, which could lead to inflammation and put the body in a pathological state. Maintaining Cu/Zn-SOD and Mn-SOD in the body at normal levels it is an important way of controlling stomach injuries [52]. As one of the key enzymes in the biological defense system, CAT antioxidant enzymes could remove oxygen free radicals, promote decomposition of H_2_O_2_ to molecular oxygen and water and also remove hydrogen peroxide in body in order to avoid the damage of H_2_O_2_ to cells and reduce tissue injuries.

Ethanol gastric mucosal injury is closely related to a decrease of gastric mucosal blood flow and lipid peroxidation induced by oxygen free radicals [53]. By strengthening the removal of free radicals, keeping the activity of active enzymes such as Cu/Zn-SOD, Mn-SOD and CAT in body is an important way of avoiding ethanol gastric mucosal injury [54].

## 3. Experimental Section

### 3.1. Shuidouchi Fermentation

Five kilograms of dry soybeans were soaked in 12.5 L distilled water for 12 h, and cooked under 120 °C for 1 h. then the water was removed and the cooked soybeans were equally divide into five parts, which were placed in ceramic, plastic, metal and glass vessels covered by gauze in a constant temperature incubator at 45 °C so natural fermentation could occur for 72 h. After fermentation, soybeans were cooled, dried and crushed to extract the soybean isoflavones. 

### 3.2. Isoflavone Determination

Two kilograms of freeze-dried fermented soybean powder were Soxhlet extracted with 32 L 70% aqueous ethanol solution for 6 h, and then the distilled ethanol was added with 5 mol/L HCl and N-503. The mixture was kept in a water bath at 30 °C for hydrolysis and extraction of soybean isoflavones for 2 h. Stratification in the separating funnel also takes up the N-503 layer. NaOH (5 mol/L) solution was added for back extraction, then the water layer was added with hydrochloric acid after stratification to precipitate soy isoflavones. After centrifugation, the sediment was washed to a neutral state and freeze-dried to extract soybean isoflavones. Taking daidzein and genistein as standards, the absorbance of different concentrations of daidzein and genistein were determined at 260 nm, and a standard curve of soybean isoflavone concentration was plotted for the content of isoflavones in soybeans [4].

### 3.3. Mice Experiment

Mice for this experiment were divided into seven groups, including normal group, control group, ceramic vessel fermented Shuidouchi (CVFS) group, plastic vessel fermented Shuidouchi (PVFS) group, metal vessel fermented Shuidouchi (MVFS) group, glass vessel fermented Shuidouchi (GVFS) group and ranitidine group, having 10 mice in each group. During the first 14 d, mice in the normal group and the control group were gavaged with 0.2 mL distilled water once a day, while mice in the other groups were gavaged 0.2 mL of Shuidouchi extract with the concentration of 500 mg/kg once. Mice in the drug treatment group were gavaged 0.2 mL ranitidine with a concentration of 50 mg/kg. From the 14th day, all mice were cut off food, but allowed to drink water freely. In addition to mice in the normal group, all mice in other group were gavaged a stomach injury inducer (0.1 mL HCl/ethanol/10 g body weight, 60% in 150 mM HCl) after 24 h and then killed after 1 h [8]. Heart blood was taken for centrifugal separation (4000 r/min, 10 min), where the upper serum was kept and the stomach was anatomized for further use. The experiments were performed following a protocol approved by the Animal Ethics Committee of Chongqing Medical University (Chongqing, China).

### 3.4. Mice Gastric Mucosal Injury Evaluation 

The gastric secretion volume of mice were determined with a 10 mL measuring cylinder, and the pH of gastric juice of mice were determined using a SevenEasy pH meter (Mettler Toledo, Schwerzenbach, Switzerland). The isolated stomachs were inflated by injecting 1% formalin solution (10 mL) for 10 min to fix the tissues, and opened along the greater curvature. The area (mm^2^) of hemorrhagic lesions that had developed in the stomach was measured under a Leica MZ7.5 dissecting microscope (Leica, Bensheim, Germany) with a square grid.

### 3.5. Mice Serum Levels Measurement

Serum MTL, Gas, SS and VIP levels were determined with radioimmunoassay kits (Beijing Puer Weiye Biotechnology Co., Ltd., Beijing, China) according to the manufacturer’s protocols.

### 3.6. Mice Cytokine IL-6, IL-12, TNF-α and IFN-γ Levels Measurement

Serum IL-6, IL-12, TNF-α and IFN-γ levels were measured with a commercial ELISA kit (ELISA MAX, Biolegend, San Diego, CA, USA) according to the manufacturer’s protocol.

### 3.7. Gastric Tissues SOD, NO and MDA Activities Measurement

Gastric tissues SOD, NO and MDA activities were determined with appropriate assay kits (Nanjing Jiancheng Bioengineering Institute, Nanjing, Jiangsu, China) according to the manufacturer’s protocols.

### 3.8. mRNA Expression Determination (RT-PCR Assay)

Stomach tissues in mice were shattered by an ultrasonic pulverizer and RNA was extracted using RNAzol reagent. RNA extract of stomach tissues was diluted to 1 μg/μL. oligodT18 (1 μL), RNase, dNTP with MLV enzymes and 10 μL 5 × buffer were added into 2 μL RNA extraction of stomach tissues to synthesize cDNA under the conditions of 37 °C for 120 min, 99 °C for 4 min, 4 °C for 3 min. Then the expressions were amplified by the reverse transcription-polymerase chain reaction method, while house-keeping gene GAPDH was taken as reference. Agarose gel (1%) with ethidium bromide was used for electrophoresis to check the PCR amplification products [8].

### 3.9. Statistical Analysis

Experimental data were presented as mean ± standard deviation (SD). Differences between the mean values for individual groups were assessed by one-way analysis of variance (ANOVA) with Duncan’s multiple range test. *p* < 0.05 was considered to indicate a statistically significant difference. SAS version 9.2 (SAS Institute Inc., Cary, NC, USA) was used to conduct the statistical analyses.

## 4. Conclusions 

Through molecular biology methods, this research built stomach injury animal models to study the effect on inhibition of stomach injuries when Shuidouchi fermented in different vessels was administered to mice. By analyzing the content of soybean isoflavones, the results showed that the glass vessel was more advantageous for fermentation, producing more soybean isoflavones. These isoflavones had functional effects, which could cause molecular changes in mice bodies, as inflammation and oxidation factors were changed by Shuidouchi. Shuidouchi could inhibit the inflammation factors and increase the antioxidation factors. By further analyzing animal serum and tissues, using glass vessel to ferment Shuidouchi could decrease the MTL, Gas serum levels and increase the SS, VIP serum levels compared with Shuidouchi fermented in other vessels and no Shuidouchi treatment for control mice. The Shuidouchi fermented in glass vessel could also better lower cytokine levels (IL-6, IL-12, TNF-α and IFN-γ) in stomach injury of mice, increase the content of SOD, NO and reduce the content of MDA in mice gastric tissues. By further analyzing mRNA in related genes in stomach tissues with RT-PCR experimental technology at the molecular level, it had been found out that Shuidouchi could improve the strength of expression of IκB-α, EGF, EGFR, nNOS, eNOS, Mn-SOD, Gu/Zn-SOD, CAT in stomach-injured mice tissues and reduce the expression strength of NF-κB, COX-2, iNOS. The effect of Shuidouchi fermentation in glass vessels was more intense, which was significantly different from that of Shuidouchi produced in other kinds of vessels. 
